# Mixture Optimization of Cementitious Materials Using Machine Learning and Metaheuristic Algorithms: State of the Art and Future Prospects

**DOI:** 10.3390/ma15217830

**Published:** 2022-11-06

**Authors:** Yaxin Song, Xudong Wang, Houchang Li, Yanjun He, Zilong Zhang, Jiandong Huang

**Affiliations:** 1 Lijiahao Coal Mine, Baotou Energy Co., Ltd., China Energy Investment Corporation, Hantai Town, Dongsheng District, Erdos 017008, China; 2 College of Resources, Shandong University of Technology, Qiangangwan Road, Huangdao District, Qingdao 100027, China; 3 School of Civil Engineering, Guangzhou University, Guangzhou 510006, China

**Keywords:** cementitious materials, mixture optimization, machine learning, metaheuristic algorithms, MOO, BAS

## Abstract

The hybrid optimization of modern cementitious materials requires concrete to meet many competing objectives (e.g., mechanical properties, cost, workability, environmental requirements, and durability). This paper reviews the current literature on optimizing mixing ratios using machine learning and metaheuristic optimization algorithms based on past studies on varying methods. In this review, we first discuss the conventional methods for mixing optimization of cementitious materials. Then, the problem expression of hybrid optimization is discussed, including decision variables, constraints, machine learning algorithms for modeling objectives, and metaheuristic optimization algorithms for searching the best mixture ratio. Finally, we explore the development prospects of this field, including, expanding the database by combining field data, considering more influencing variables, and considering more competitive targets in the production of functional cemented materials. In addition, to overcome the limitation of the swarm intelligence-based multi-objective optimization (MOO) algorithm in hybrid optimization, this paper proposes a new MOO algorithm based on individual intelligence (multi-objective beetle antenna search algorithm). The development of computationally efficient robust MOO models will continue to make progress in the field of hybrid optimization. This review is adapted for engineers and researchers who want to optimize the mixture proportions of cementitious materials using machine learning and metaheuristic algorithms.

## 1. Introduction

To optimize the mixture proportions of cementitious materials, a large number of trial batches are usually prepared in a laboratory [1]. The laboratory-based method is time, labor, and resource intensive if multiple objectives of cementitious materials need to be optimized, considering a large number of influencing variables under highly non-linear constraints. As the basic mechanical objective, uniaxial compressive strength (UCS) must be considered to evaluate the stability of structures against loads during the design process of mixture proportions. Another necessary objective of cementitious materials is the cost which has enormous economic implications in construction projects requiring large volumes of concrete [2,3]. If many objectives (e.g., slump, CO_2_ emission, density, durability) need to be considered, the number of samples that need to be prepared in a laboratory may increase exponentially. Furthermore, many factors will influence the generalization ability of the results. These factors include constituent characteristics (e.g., grading, shape and size of aggregates, chemistry, and type of cementitious materials) and spatiotemporal environmental variability (humidity and temperature) [4,5]. In addition, the experimental based methods can yield well-performing proportions of mixtures rather than best-performing ones, as only a limited number of samples can be prepared in the laboratory.

To overcome the limitations of laboratory-based mixture optimization, an alternative solution is to use computational optimization methods based on machine learning (ML) and metaheuristic optimization algorithms [6,7]. This method firstly models the objectives of cementitious materials using ML approaches and then searches for optimal mixture proportions using metaheuristic optimization algorithms. ML models can accurately model the objectives of cementitious materials without knowing the explicit relationships between the objectives and the input variables [8], which is superior to conventionally used linear or nonlinear regression models that rely highly on the coefficients of the models [9,10]. Currently, the widely used ML models for modeling objectives include the Artificial Neural Network (ANN), Support Vector Machine (SVM), and tree-based models, such as Decision Tree (DT), Random Forest (RF), and Gradient Boosted Regression Tree (GBRT) [11,12,13].

After modeling objective functions, optimization algorithms are then applied to search for optimal mixture proportions that satisfy multiple competing objectives. Generally, there are two main types of optimization algorithms: stochastic and deterministic [14]. For the same starting point, deterministic algorithms will generate the same set of solutions, while stochastic algorithms will produce different ones. Therefore, deterministic algorithms may be trapped in local optima in the process of searching. A solution is to use stochastic algorithms that are comprised of a random component and a deterministic component. Many forms can be used for the stochastic component such as random walks and randomly sampling the searching space. Using these approaches, stochastic algorithms can then jump out of the locality. The most widely used stochastic algorithms are swarm-intelligence based metaheuristic algorithms such as Particle Swarm Optimization (PSO) [15] and Genetic Algorithm (GA) [16]. These two algorithms have been widely used in the single-objective mixture optimization of cementitious materials [4]. For multi-objective mixture optimization, a single best solution does not exist, and hence these swarm-based metaheuristic algorithms must be extended into multi-objective optimization (MOO) versions to approximate the true Pareto front of the MOO problems. Widely used MOO algorithms include Multi-Objective Particle Swarm Optimization (MOPSO) [17], Multi-Objective Differential Evolution (MODE) [18], and the Multi-Objective Genetic Algorithm (MOGA) [19]. Besides swarm-intelligence based algorithms, new individual-intelligence based algorithms, such as the Beetle Antennae Search (BAS) algorithm with higher searching efficiency have also been applied to concrete mixture optimization.

This paper firstly reviews the traditional mixture design methods in Section 2, and then elaborates on the three steps of mixture optimization using ML models and metaheuristic algorithms, i.e., (1) problem formulation, (2) objective modeling, and (3) optimization. Finally, the prospects for mixture optimization of cementitious materials are discussed in Section 4.

## 2. Conventional Methods of Mixture Optimization of Cementitious Materials

### 2.1. Experimental-Based Design Methods

#### 2.1.1. Prescriptive-Based Approach

**Figure 1:**
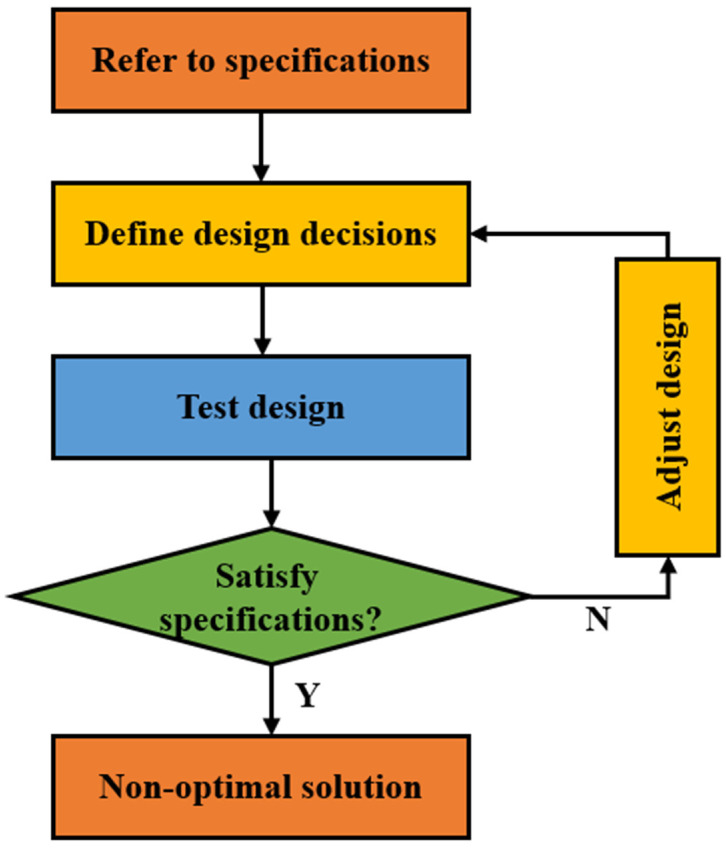
Prescriptive-based approach.

The prescriptive-based approach is a step-by-step approach that is widely used to proportion mixtures of cementitious materials [20]. This method has evolved from the arbitrary volumetric method (cement: fine aggregate: coarse aggregate = 1:2:3) of the early 1990s [21] to the presently used absolute volume method (ACM) and weight method developed by the Portland Cement Association [22] and the American Concrete Institute [23]. The weight method uses the known weight of the cementitious materials per unit volume to proportion the mixtures. Although simple, it is not as accurate as ACM which calculates the absolute volume of each ingredient in a unit volume of the cementitious materials. The prescriptive specifications usually incorporate requirements for the mixture composition such as type of cement and aggregate, limits on the content of cement and admixtures, minimum or maximum water-to-binder ratio, etc. The prescriptive-based approach is demonstrated in Figure 1.

The main advantage of the prescriptive-based approach is that the mixture proportions are directed by the approach itself. The producer is not liable for the durability, strength, or cost of the cementing material as long as it meets the strict prescribed requirements. Therefore, this method can be applied in rural areas where concrete producers are not veterans in designing mixture proportions [24]. However, this method is limited by the lack of flexibility for the contractor or producer for tailoring mixture proportions, which may lead to unsatisfactory performance and higher costs for cementitious materials [24].

#### 2.1.2. Performance-Based Approach

**Figure 2:**
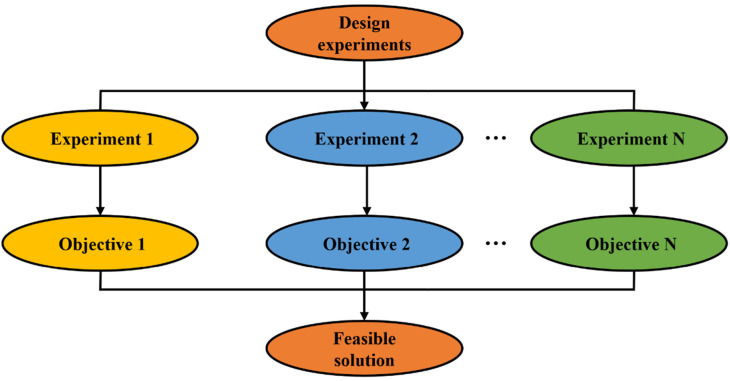
Performance-based approach.

Compared with the prescriptive-based concrete mixture design methods, the performance-based methods proportion mixtures of cementitious materials from trail batches in the laboratory to satisfy design specifications without strict requirements for amounts and ratios of constituents of concrete [25,26]. To this end, the producer can choose an arbitrary amount of water, cement, supplementary material, and fine and coarse aggregates by trial-and-error methods to achieve the desired compressive strength, durability, or cost. The prescriptive-based approach is demonstrated in Figure 2.

However, the number of samples that need to be prepared may increase exponentially when multiple objectives for the cementitious materials (e.g., cost, strength, durability, etc.) are required to be optimized, or several influential parameters (ingredients) are considered in proportioning mixtures of cementitious materials [27]. Furthermore, this method can yield only one feasible design solution with an iterative design process rather than optimal (best-performing) solutions [27]. 

### 2.2. Taguchi Method

To reduce the number of samples to be prepared using traditional experimental design methods, the Taguchi method was proposed [28]. This optimization method can not only minimize the cost of concrete but also minimize the variability of the targets by minimizing the effects of uncontrollable factors. This laboratory-based optimization method can also derive optimal work-flow conditions [29].

**Figure 3:**
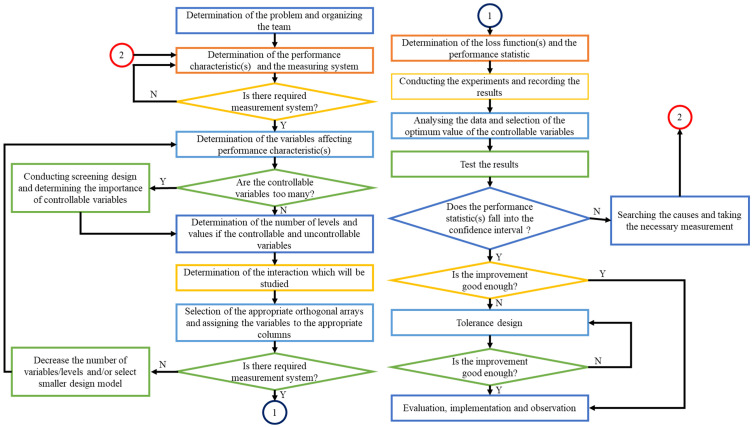
Flowchart of the systematic Taguchi method [31].

The Taguchi method optimizes one or multiple performance properties using the Quantity Design Method. A systematic Taguchi method is illustrated in Figure 3. The steps can be iterated as follows [30]:

1. Ascertain the performance characteristic and choose factors to be assessed;
2. Decide on the likely relationship between factors and the number of quantity levels;
3. Choose the suitable orthogonal array for the factors;
4. Perform experiments on the basis of the arrangement of the orthogonal array;
5. Compute the statistics of performances;
6. Obtain the results of experiments by using analysis of variance (ANOVA);
7. Choose optimum levels of factors;
8. Validate the optimum factors by conducting confirmation experiments.

The optimization criteria are represented by the performance characteristics which can be classified into three types: the nominal-the-better, the smaller-the-better, and the larger-the-better, as follows.

The nominal-the-better:

(1) $$\frac{S}{N}=-10\log_{10}\left(\frac{1}{n}\sum_{i=1}^{n}{\left(Y_{i}-Y_{0}\right)}^{2}\right)$$

where *S*/*N* is the performance statistics; *n* is the number of repetitions for an experimental combination; *Y_i_* is the performance value of the *i*th experimental and *Y*_0_ is the nominal value desired.

The smaller-the-better:

(2) $$\frac{S}{N}=-10\log_{10}\left(\frac{1}{n}\sum_{i=1}^{n}Y_{i}^{2}\right)$$

The larger-the-better:

(3) $$\frac{S}{N}=-10\log_{10}\left(\frac{1}{n}\sum_{i=1}^{n}\frac{1}{Y_{i}^{2}}\right)$$

Although the Taguchi approach is very easy and straightforward to apply to mixture optimization cementitious materials without a high amount of experimentation, the influence of factors on the performance characteristic value cannot be indicated by the relative results obtained [32]. In addition, this method cannot test all the relationships between the constituents and the performances of cementitious materials since the orthogonal arrays do not include all the combinations of variables, and therefore such an experimental design may not achieve the most cost-effective solution [32].

### 2.3. Response Surface Methodology

Response surface methodology (RSM) is a statistical technique to model and analyze a process where a variety of variables influence the response of interest, and the objective of this approach is to optimize the response (the performance measure) [33]. This method can develop a generalized model using provided data samples with decreased cost and labor, is able to optimize the response based on various priorities, and can evaluate the interactions of factors.

This method has been used in mixture design since the beginning of the 21st century. In 2000, this methodology was used to optimize mixture proportions of self-consolidating concrete (SCC) considering the influence of water-to-cement ratio, contents of binder, and coarse aggregates [34]. In 2004, Bayramov et al. optimized the fracture parameters of steel fiber-reinforced concrete under the effect of two variables: the volume fraction of steel fiber and their aspect ratios, using a three-level full factorial experimental design and RSM [35]. Subsequently, this methodology has been used to optimize mixture proportions of many types of concrete such as foam concrete [36], self-compacting concrete [37], rubberized concrete [38], and high-performance concrete [39].

Generally, RSM applies a second-order model to maximize the response of the output by finding the optimal set of inputs:

(4) $$Y=\beta_{0}+\sum_{i=1}^{k}\beta_{i}x_{i}+\sum_{i=1}^{k-1}\sum_{j=i+1}^{k}\beta_{ij}x_{i}x_{j}+\sum_{i=1}^{k}\beta_{ii}x_{i}^{2}$$

where the $x_{i}$ denote the code values of the input factors (independent variables) determining the response (the output) *Y* and $\beta_{0}$, $\beta_{i}$, $\beta_{ij}$, and $\beta_{ii}\;$ are unknown parameters that can be derived using least squares regression.

Although RSM is an efficient method with the ability to solve optimization problems with a large number of design variables, two main disadvantages limit its application. The first is that RSM uses a second-order polynomial equation to model the objective function, although it is difficult to accurately obtain the regression coefficients. The second problem is that RSM only provides a locally optimal solution [40]. The first problem can be overcome by introducing ML techniques, while the second issue can be addressed using metaheuristic algorithms.

## 3. Problem Formulation of Concrete Mixture Optimization

Generally, to solve mixture optimization problems of cementitious materials, it is necessary to determine the decision variables, constraints, and objective functions of the problem.

### 3.1. Decision Variables

A decision variable is a quantity that decision-makers would like to determine. The values of decision variables will be altered to achieve optimization of the objectives [41]. In mixture optimization problems, decision variables are components whose values can be discrete or continuous. Continuous decision variables may incorporate the content of water; cement; aggregates; supplementary cementitious materials (SCMs) (e.g., fly ash, silica fume, and slag) and superplasticizer; curing age; and curing conditions (humidity and temperature), while discrete decision variables might include types of the cement; superplasticizer and SCMs; and size of aggregates. The common decision variables for mixture optimization are tabulated in Table 1.

### 3.2. Constraints

To ensure that the mixtures found by the MOO model have physical meaning, four categories of constraints should be applied as follows [42]:

(1) Range constraints

The range constraint requires that the content of decision variables should vary within a reasonable range which can be specified as per the standards or experts’ experience. The range constraint is given by:

(5) $$D_{imin}\leq D_{i}\leq D_{imax}$$

where *D_imin_* and *D_imax_* are the minimum and maximum value of the *i*-th decision variable *D_i_*, respectively.

(2) Ratio constraints

Generally, when proportioning mixtures of cementitious materials, several ratios should be constrained, such as water-to-cement ratio, superplasticizer-to-cement ratio, sand ratio, etc.

(3) Concrete volume constraint

The total volume of the components in cementitious materials are normalized to 1 m^3^. Assuming that the cementitious materials are made up of cement, water, coarse and fine aggregates, and superplasticizer, the volume constraint is given by

(6) $$V_{m}=\frac{Q_{c}}{U_{c}}+\frac{Q_{w}}{U_{w}}+\frac{Q_{fa}}{U_{fa}}+\frac{Q_{ca}}{U_{ca}}+\frac{Q_{sp}}{U_{sp}}$$

where $Q_{c}$, $Q_{w}$, $Q_{fa}$, $Q_{ca}$, and $Q_{sp}$ denote the weight of cement, water, fine aggregate, coarse aggregate, and superplasticizer respectively. *U_c_*, *U_w_*, *U_fa_*, *U_ca_*, and *U_sp_* are the unit weight of cement, water, fine aggregate, coarse aggregate, and superplasticizer, respectively.

### 3.3. Objective Functions

Statistical and ML models are widely used to model objective functions for mixture optimization of cementitious materials. 

#### 3.3.1. Statistical Model

The linear regression model that establishes the relationship between water-to-cement (*w*/*c*) ratio and the UCS of concrete is widely used [43]:

(7) $$f_{c}=b_{0}+b_{1}\cdot w/c$$

where $f_{c}$ is the property of concrete; $b_{0}$ and $b_{1}$ are coefficients. Since many other variables also influence concrete properties, a multivariable linear regression model was developed [44]:

(8) $$f_{c}=b_{0}+b_{1}\cdot x_{1}+b_{2}x_{2}+\cdots b_{n}x_{n}$$

where $x_{1},\;x_{2},\ldots x_{n}$ are influencing variables such as *w*/*c*, the content of cementitious materials, the content of aggregates, curing age, etc. 

It is known that the relationship between the influencing variables and the properties of cementitious materials is highly nonlinear, and hence the prediction accuracy of the above linear models is not high [45]. To address this issue, a multivariable power equation has been introduced as follows [46]:

(9) $$f_{c}=b_{0}x_{1}^{a_{1}}x_{2}^{a_{2}}x_{3}^{a_{3}}\ldots x_{n}^{a_{n}}$$

where the values of $a_{1}\ldots a_{n}$ are determined from regression analysis of the statistical data. 

The statistical models are able to establish the complex nonlinear relationships between the influencing variables and the properties of cementitious materials; however, they cannot model the random noise in the data. In addition, it is difficult to accurately obtain the regression coefficients in these models. These limitations can be overcome by ML models.

#### 3.3.2. Machine Learning Models

Machine learning (ML) is a computational method that can identify complicated and meaningful patterns from data. ML can facilitate decision-making intelligently without knowing the equations between inputs and outputs in advance [47]. The development of an ML model includes defining a representation of the target function and developing a model to learn the target function by training the given data samples. ML is classified into unsupervised, semi-supervised, supervised, and reinforced learning [48]. In concrete mixture optimization, supervised learning is widely used [4]. In supervised learning, the data are labeled (i.e., the outputs of the data are known). In the training process, the parameters of an algorithm are adjusted by comparing the actual output values with the predicted ones. The training process stops when the error between the actual and output values reaches the threshold. The supervised learning task can be further classified into regression if the target variable is continuous and classification if the variables are discrete. To find patterns in data, several ML models can be used. However, based on the “no free lunch” theorem of ML, we cannot find a single ML model that universally behaves best for every dataset [49]. Therefore, a comparative study should be conducted to select the winning ML model for modeling objectives of cementitious materials for the optimization procedure. Below, we will introduce several ML models that are widely used for modeling concrete mechanical properties and durability.

Despite the extensive research on the modeling objectives of cementitious materials using ML models, there still exist two main gaps in this field [12]. First, the previous research does not indicate a grounding in the best-performed approaches of the ML models. A pipeline of approaches is generated by the standard procedure in ML which increases the complexity of the procedure. Second, consensus on the best model architectures for modeling objectives of concrete in large datasets with various noisy parameters has not yet been reached.

##### Artificial Neural Network

**Figure 4:**
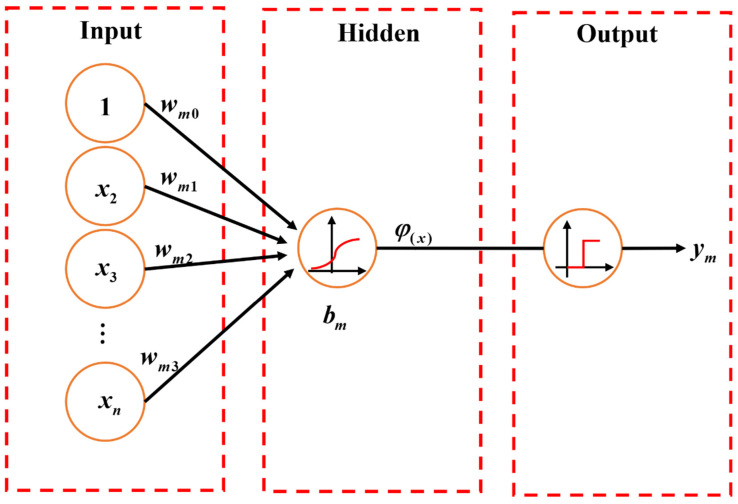
Architecture of ANN.

An artificial neural network (ANN) is inspired by the function and structure of biological neural networks. ANN is extensively used to capture highly nonlinear patterns of large-scale data in many fields [50]. In the architecture of ANN (Figure 4), artificial neurons constitute the input, hidden, and output layers. The signal or information propagates from the input layer, through the hidden layer to the output layer in the form of weights as follows:

(10) $$y_{m}=\varphi \left(\sum_{i=0}^{n}w_{mi}x_{i}\right)+b_{m}$$

where $x_{i}$ is the input signal of a neuron; $w_{mi}$ is the weight assigned to the corresponding input neuron; $y_{m}$ is the output signal; $b_{m}$ is the bias value of the *m*th output neuron; φ is the activation function that is applied to transform the output value into the design range. For instance, the Sigmoid activation function can map the output values between 0 to 1 or −1 to 1. Other commonly used activation functions include rectified linear unit and hyperbolic activation functions.

ANN can be classified into different types according to different learning methods and architecture types, such as single-layer or multilayer networks as per the number of hidden layers, feedforward or recurrent networks based on the connection pattern, and adaptive or fixed neural networks according to the adjustment nature of weights. In the training process of ANN, the weight and bias values are adjusted to minimize the error between predicted and actual output values. Commonly used training methods include the self-organizing map, the popular back-propagation algorithm, and the real-coded genetic algorithm [51].

Among ML models for predicting the objectives of concrete, ANN dominates the literature. Many types of neural networks have been applied. ANN was firstly used to model the strength of high-performance cementitious materials by Yeh [52]. Then several other types of ANN were developed to model the properties of cementitious materials. Dias and Pooliyadda used a Backpropagation Neural Network (BPNN) to predict the strength and slump of ready-mixed cementitious materials and high-strength concrete [53]. BPNN achieved higher prediction accuracy than multiple regression because BPNN has a higher generalizing ability to tolerate errors. In addition, BPNN can apply gradient methods to train multilayer networks, and update weights to minimize loss. However, BPNN requires more computational time to train the networks. Moreover, BPNN obtains deterministic rather than probabilistic results. To overcome this issue, the Probabilistic Neural Network (PNN) was developed by Lee et al. to predict concrete strength [54]. The dynamic decay adjustment algorithm was used to automatically calculate the smoothing parameter of PNN without external engineering judgment. 

Another promising method is the fuzzy neural network conjunction model. Zarandi et al. predicted the compressive strength of cementitious materials by developing the Fuzzy Polynomial Neural Network (FPNN) [55]. The proposed FPNN was a combination of the PNN and the Fuzzy Neural Network and provided strong predictions of concrete strength. To reduce the noise of training data, ensemble models based on bagging and boosting were investigated by Erdal et al. [56]. The prediction accuracy of ANN ensembles was then enhanced by coupling with discrete wavelet transform. Despite the wide application of ANN in the prediction of properties of cementitious materials, ANN has inherent shortcomings, such as slow convergence and trapping in local optima. This is caused by randomly initializing the bias and weight values of the network before training. To address this issue, swarm intelligence based metaheuristic optimization algorithms such as the Genetic Algorithm (GA) [57,58] and the Particle Swarm Optimization (PSO) [59,60] have been used to search for the optimal parameters of ANN. 

##### Support Vector Machine

The support vector machine (SVM) is one of the popular types of supervised ML algorithms. It learns the complex relationships between inputs and outputs by using kernel tricks to map the training data into a higher dimensional characteristic space [61]. SVM maximizes the separation between the training data and the hyperplane to minimize the upper bound of errors. SVM can be used to solve classification and regression problems. For modelling concrete objectives, support vector regression (SVR) is usually applied. The formulation of SVR is introduced as follows.

A regression equation can be defined as

(11) f(x)=w·φ(x)+b

where each **x** is an *l*-dimensional input variable; **w** is the weight vector; φ(x) denotes a nonlinear mapping function; *b* represents the bias value. The degree of deviation between the actual output **y***_i_* and the predicted output $f\left(x_{i}\right)$ can be measured by the following loss function

(12) $$ℒ\left(x,y,f\right)={\left|y_{i}-f\left(x_{i}\right)\right|}_{ℰ}=\{\begin{array}{ll} 0, & \left|y_{i}-f\left(x_{i}\right)\right|<\varepsilon \\ \left|y_{i}-f\left(x_{i}\right)\right|-\varepsilon , & \left|y_{i}-f\left(x_{i}\right)\right|\geq \varepsilon \end{array}$$

If the deviation is smaller than the largest tolerance error ε for each **x***_i_*, f(x) will be obtained. It can be seen from the above function that the data within the ε-tube will not be penalized. The support vectors for building f(x) are the data that are located outside or on the ε-tube.

The problem aims to minimize w and *b* by introducing the structural risk minimization [62]:

(13) $$ℛ\left(w\right)=\frac{1}{2}{\left\|w\right\|}^{2}+\sum_{i=1}^{n}ℒ\left(x,y,f\right)$$

**Figure 5:**
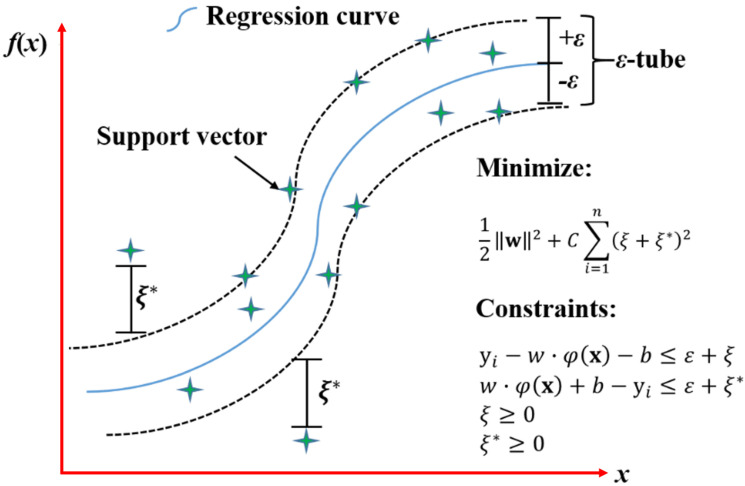
Example of nonlinear SVR with ε-tube.

Slack variables $\xi_{i}$ and $\xi_{i}^{*}$ are applied to allow some errors. The above function can then be rewritten as

$$min_{w,b,\;\xi ,\xi^{*}}ℛ\left(w\right)=\frac{1}{2}{\left\|w\right\|}^{2}+C\sum_{i=1}^{n}\left(\xi_{i}+\xi_{i}^{*}\right)$$

(14) $$\{\begin{array}{c} y_{i}-w\cdot \varphi \left(x\right)-b\leq \varepsilon +\xi_{i}\\ w\cdot \varphi \left(x\right)+b-y_{i}\leq \varepsilon +\xi_{i}^{*}\\ \xi_{i}\geq 0\\ \xi_{i}^{*}\geq 0 \end{array}$$

where *C* is a penalty parameter that determines the trade-off between the penalizing term ‖w‖ and the training error. An example of nonlinear SVR with an *ε*-tube is shown in Figure 5.

Lagrange multipliers can be used to solve the problem:

$$L\left(w,b,\xi ,\alpha ,,\mu \right)=\frac{1}{2}{\left\|w\right\|}^{2}+C\sum_{i=1}^{n}\left(\xi_{i}+\xi_{i}^{*}\right)$$

$$-\sum_{i=1}^{n}\alpha_{i}\left(\varepsilon +\xi_{i}-y_{i}+w\cdot \varphi \left(x_{i}\right)+b\right)$$

$$-\sum_{i=1}^{n}\alpha_{i}^{*}\left(\varepsilon +\xi_{i}^{*}+y_{i}-w\cdot \varphi \left(x_{i}\right)-b\right)$$

(15) $$-\sum_{i=1}^{n}\left(\mu_{i}\xi_{i}+\mu_{i}^{*}\xi_{i}^{*}\right)$$

where $\alpha_{i}\geq 0$, $\alpha_{i}^{*}\geq 0$, $\mu_{i}\geq 0,$ and $\mu_{i}^{*}\geq 0$ are Lagrange multipliers. KKT functions should be satisfied to solve the constraint functions with strong duality (the primal optimal objective and the dual optimal objective are equal) as follows [63]

(16) $$\{\begin{array}{c} \frac{\partial L}{\partial w}=w-\sum_{i=1}^{n}\left(\alpha_{i}-\alpha_{i}^{*}\right)\varphi \left(x_{i}\right)=0\\ \frac{\partial L}{\partial b}=\sum_{i=1}^{n}\left(\alpha_{i}-\alpha_{i}^{*}\right)=0\\ C-\alpha_{i}-\mu_{i}=0\\ C-\alpha_{i}^{*}-\mu_{i}^{*}=0 \end{array}$$

To find the optimal solution, the product of dual variables and constraints is 0:

(17) $$\{\begin{array}{c} \alpha_{i}\left(\varepsilon +\xi_{i}-y_{i}+w\cdot \varphi \left(x_{i}\right)+b\right)=0\\ \alpha_{i}^{*}\left(\varepsilon +\xi_{i}^{*}+y_{i}-w\cdot \varphi \left(x_{i}\right)-b\right)=0\\ \left(C-\alpha_{i}\right)\xi_{i}=0\\ \left(C-\alpha_{i}^{*}\right)\xi_{i}^{*}=0 \end{array}$$

The Lagrange dual problem can be obtained after solving the above equations:

$$\underset{i}{\max}\left(-\frac{1}{2}\sum_{i=1}^{n}\sum_{j=1}^{n}\left(\alpha_{i}-\alpha_{i}^{*}\right)\left(\alpha_{j}-\alpha_{j}^{*}\right)x_{i}^{T}x_{j}-\varepsilon \sum_{i=1}^{n}\left(\alpha_{i}+\alpha_{i}^{*}\right)+\sum_{i=1}^{n}y_{i}\left(\alpha_{i}-\alpha_{i}^{*}\right)\right)$$

(18) $$s.t.\{\begin{array}{c} \sum_{i=1}^{n}\left(\alpha_{i}-\alpha_{i}^{*}\right)=0\\ \alpha_{i},\alpha_{i}^{*}\epsilon \left[0,C\right] \end{array}$$

By replacing w with $\sum_{i=1}^{n}\left(\alpha_{i}-\alpha_{i}^{*}\right)\varphi \left(x_{i}\right)$, the regression function is given by

(19) $$f\left(x\right)=\sum_{i=1}^{n}\left(\alpha_{i}-\alpha_{i}^{*}\right)\varphi \left(x_{i}\right)x+b$$

SVM has been used to predict the compressive strength of lightweight aggregate concrete [64] and the elastic modulus of high and normal strength cementitious materials [65]. In addition, the performance of SVM in the prediction of the properties of cementitious materials has been compared with ANN. Akande et al. modelled the UCS of cementitious materials using ANN and SVM, and found that the presence of local minima has more influence on ANN [66]. Sonebiet al. [47] predicted fresh properties (e.g., v-funnel time, L-box, and slump flow) of self-compacting concrete using SVM. The results indicated that SVM achieved higher prediction accuracy than ANN when predicting complex fresh properties (such as slump) of cementitious materials [67].

While SVM is popular in the prediction of the properties of cementitious materials, it faces several inherent drawbacks. Firstly, the performance of SVM is significantly affected by the selection of the penalty parameter and the kernel parameter. Second, the decision surface is determined by considering the training points equally. These limitations can be overcome by proposing some other types of SVM. Cheng et al. used a weighted Support Vector Machine (WSVM) to predict the compressive strength of high-performance concrete [68]. WSVM obtains the decision surface by considering the weights of different training data, and hence achieves higher prediction accuracy compared with traditional SVM. Pham et al. used a Least Squared Support Vector Machine (LSSVM) to predict the compressive strength of high-performance concrete. The method achieved the most desirable performance with low prediction errors in comparison with other ML models [69]. Generally, multiple properties need to be considered for a specific concrete (e.g., the permeability and UCS of pervious concrete). Zhang et al. first introduced the multi-output least squares support vector machine (MOLSSVM) to predict the permeability and UCS of pervious concrete. The proposed MOLSSVM was able to utilize the relationship between UCS and permeability and thus achieved higher prediction accuracy than single-output models [70].

##### Tree-Based Models

(1) Decision tree

The Decision Tree (DT) is a widely employed algorithm for both classification and regression problems due to its rule induction, representation simplicity, and better accuracy [71]. DT was initially developed for solving classification problems, e.g., C4.5 [72] and Dichotomiser 3 [73]. Then, regression problems can be solved by extending DT to create the Classification and Regression Tree (CART) [74]. A DT is able to decompose/transform a complex problem into smaller ones by using a series of “if-then” statements. Therefore, after the efficient learning process of DT, the answers are provided in a simple symbolic representation. The structure of a DT is like a flow chart. A “test” on an attribute is represented by an inter-node. The outcome of the test is represented by a branch, and a class label is represented by a leaf node. 

In the process of growing a DT, the samples are split by selecting one of the input variables. The internal node (a “test” on an attribute) is split into subsequent nodes by selecting the best split point. The input space is divided such that smaller errors between the actual and predicted outputs are achieved. The predicted outputs ${\hat{y}}_{i}$ for a regression tree at the *i*th leaf node are determined as follows

(20) $${\hat{y}}_{i}=\frac{\sum_{j\in t_{i}}y_{j}}{\left|t_{i}\right|}$$

where $t_{i}$ represents the *i*-th leaf node; $\left|t_{i}\right|$ is the number of instances at the *i*-th leaf node; $y_{j}$ denotes the *j*-th actual output. The least squares deviation (LSD) impurity measure is used as the splitting criterion [75]:

(21) $$I\left(t_{i}\right)=\sum_{j\in t_{i}}{\left(y_{j}-{\hat{y}}_{i}\right)}^{2}$$

where $I\left(t_{i}\right)$ denotes the impurity measure (a measure of the homogeneity of the labels at the node) at the *i*-th node. The splitting criterion is calculated based on LSD:

(22) $$\Delta I=I\left(t_{p}\right)-P_{l}I\left(t_{l}\right)-P_{r}I\left(t_{r}\right)$$

where $t_{p}$, $t_{l},$ and $t_{r}$ represent the parent model, left-child node and right-child node, respectively. $P_{r}$ and $P_{l}$ denote the proportions of instances given to the right and left nodes, respectively. By maximizing ΔI, the split point is obtained.

DT has higher prediction accuracy when dealing with categorical variables in comparison with other regression models, as DT predicts an output value as per the induced rules without defining the distance measures for categorical variables or requiring the conversion of categorical variables.

(2) Random Forest

**Figure 6:**
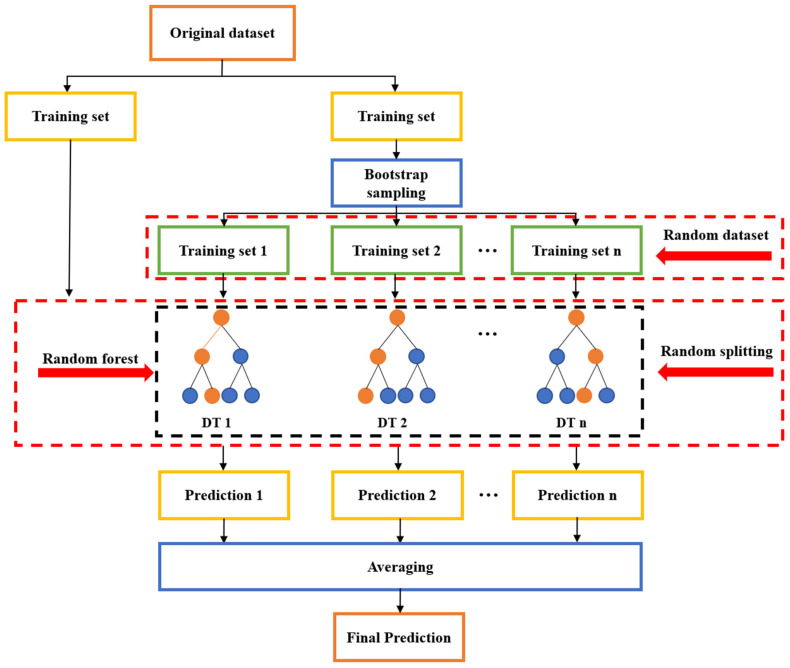
Construction of RF.

The Random Forest (RF) algorithm is a popular ensemble algorithm which employs random split selection and the bagging method to build an uncorrelated forest of trees [76]. To train RF, the training set *S_n_* is randomly split into a number of subsets and in each subset a de-correlated DT is grown. The DTs are then combined into RF with the use of bagging. A subset containing *n* samples with the selective probability of 1/*n* is called a bootstrap sample $S_{n}^{\Theta }$, where Θ denotes an independently distributed vector. Assuming that *m* DTs are generated from *m* bootstrap samples ($S_{n}^{\Theta_{1}},S_{n}^{\Theta_{2}},\ldots ,S_{n}^{\Theta_{m}}$), *m* outputs are then obtained: ${\hat{Y}}_{1}=\hat{h}\left(X,S_{n}^{\Theta_{1}}\right),{\hat{Y}}_{2}=\hat{h}\left(X,S_{n}^{\Theta_{2}}\right),\ldots ,{\hat{Y}}_{m}=\hat{h}\left(X,S_{n}^{\Theta_{m}}\right)$. The final output of RF is the mean value of the *m* DT outputs, i.e., $\hat{Y}=\sum_{i=1}^{m}\hat{h}\left(X,S_{n}^{\Theta_{i}}\right)$. A flowchart of the construction of RF is shown in Figure 6.

(3) Gradient Boosted Regression Tree

A Gradient Boosted Regression Tree (GBRT) trains the model using least-squares regression. For each iteration *m*, the response $\hat{y}$ is predicted by a weak model $F_{m}$. Then, an estimator *h* is introduced to improve the prediction of the model:

(23) $$F_{m+1}\left(x\right)=F_{m}\left(x\right)+h\left(x\right)$$

where $F_{m}$ represents the GBRT model with *m* DTs. For each boosting iteration *m*, a new DT is introduced to the GBRT. Term *h* is given by [77,78,79]:

(24) $$F_{m+1}\left(x\right)=F_{m}\left(x\right)+h\left(x\right)=y$$

(25) $$h_{m}\left(x\right)=y-F_{m}\left(x\right)$$

**Figure 7:**
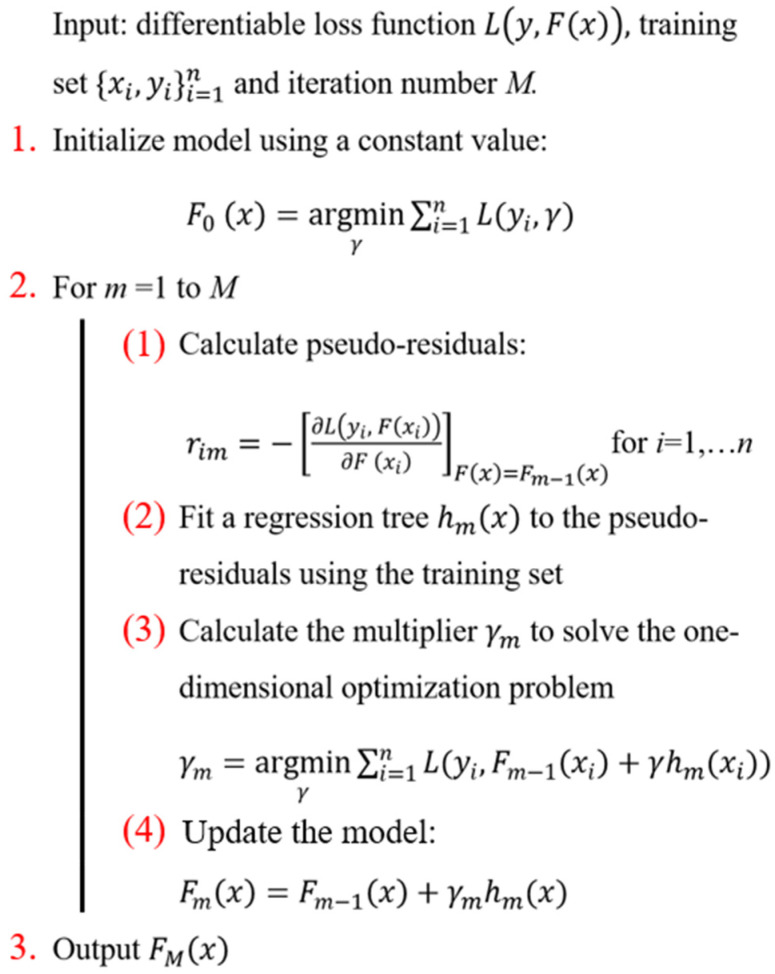
Pseudocode of GBRT [80].

Therefore, gradient boosting will fit *h* to the residual $y-F_{m}\left(x\right)$. The pseudocode of the gradient boosting method is shown in Figure 7.

(4) Modelling properties of cementitious materials using tree-based models

The M5 model tree algorithm is a popular DT algorithm for predicting the properties of cementitious materials. Some researchers indicate that the M5 tree algorithm is more understandable than ANN and more accurate in comparison with statistical methods [81,82]. Behnood et al. used the M5 model tree algorithm to predict the elastic modulus of cementitious materials containing recycled aggregate. Simple mathematical formula were derived using the model and this model achieved an accuracy over 80 percent above that of the other models. The M5 tree model cannot perform well in complex data-domains (e.g., dampening, logistic, or sinusoidal functions) due to its reliance on linear functions. This shortcoming can be addressed by tree-based ensemble models such as RF thanks to its capability of handling continuous as well as discrete variables while reducing variance over non-monotonic and monotonic data domains [76]. Zhang et al. employed RF regression to model the UCS of lightweight self-compacting concrete (LWSCC) [83]. The complex relationship between the UCS of LWSCC and its influencing variables was successfully modelled, and the variable importance was obtained. Another study used a metaheuristic-optimized RF model to predict the UCS of oil palm shell concrete [84]. A recently proposed Beetle Antennae Search (BAS) algorithm was modified by incorporating Levy flight and self-adaptive inertia weight to search for the hyperparameters of RF. The results show that the modified RF model achieved high prediction accuracy with a correlation coefficient of 0.9588 for predicting the UCS of oil palm shell concrete. GBRT is another tree-based ensemble model. Zhang et al. predicted the UCS and splitting tensile strength (STS) of manufactured sand concrete using three tree-based models: DT, RF, and GBRT. The results showed that GBRT achieved the highest prediction accuracy for UCS and STS with correlation coefficients of 0.99 and 0.97, respectively. The extreme boosting (XGBoost) was also used by Li et al. to predict concrete strength [85]. The hyperparameters of XGBoost were tuned by comprehension of the learning particle swarm optimizer. The results showed that the hybrid model outperformed other popular ML models in terms of accuracy and robustness. 

It is difficult to say which of the tree-based models performs best in a particular dataset. Thus, a comparison is needed when using these tree-based models for prediction of the properties of cementitious materials. In addition, as the performance of tree-based models relies on the values of their hyperparameters, it is suggested that the hyperparameters are tuned using optimization algorithms such as metaheuristic optimization algorithms.

### 3.4. Optimization Algorithms

Optimization algorithms are applied to search for optimal mixture proportions of cementitious materials with ML models as objective functions. In this regard, metaheuristic optimization algorithms are widely used due to their simple implementation and high computational efficiency [86,87]. The following section will introduce the widely used metaheuristic algorithms for mixture optimization of cementitious materials, including Particle Swarm Optimization (PSO), Genetic Algorithm (GA), and Beetle Antennae Search (BAS).

#### 3.4.1. Metaheuristic Optimization Algorithms

##### Particle Swarm Optimization

**Figure 8:**
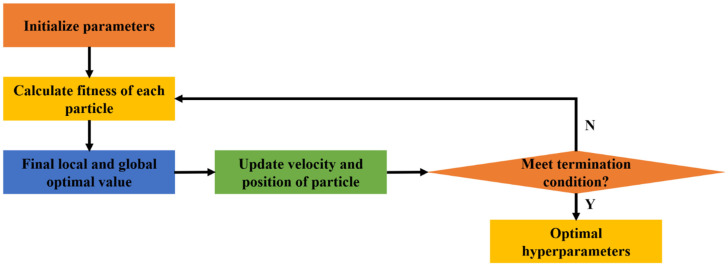
Flowchart of PSO.

The development of Particle Swarm Optimization (PSO) was inspired by the behavior of fish schooling or birds flocking [88,89]. A particle in the swarm represents a potential candidate solution to the problem. The particles move as per the best known position of themselves and the entire swarm in the search-space. The position update equation is given by

(26) $$\begin{array}{cc} v_{id}^{t+1}=w\times v_{id}^{t} & +\;c_{1}\times r_{1i}\times \left(pbest_{id}-x_{id}^{t}\right)\\ & +\;c_{2}\times r_{2i}\times \left(gbest_{id}-x_{id}^{t}\right) \end{array}$$

(27) $$x_{id}^{t+1}=x_{id}^{t}+v_{id}^{t+1}$$

where $v_{id}^{t}$ and $v_{id}^{t+1}$ represent the velocities of particle *i* at the *t*-th and (*t* + 1)-th iterations, respectively; *d* denotes the dimension of the searching space; $pbest_{id}$ and $gbest_{id}$ are the best known positions of the particle and the entire swarm, respectively; $x_{id}^{t}$ and $x_{id}^{t+1}$ represent the positions of particle *i* at the *t*-th and (*t* + 1)-th iterations, respectively; $c_{1}$ and $c_{2}$ denote acceleration coefficients; w is the initial weight; and $r_{1i}$ and $r_{2i}$ represent two random values between 0 and 1. A flowchart of PSO is illustrated in Figure 8.

##### Genetic Algorithm 

**Figure 9:**
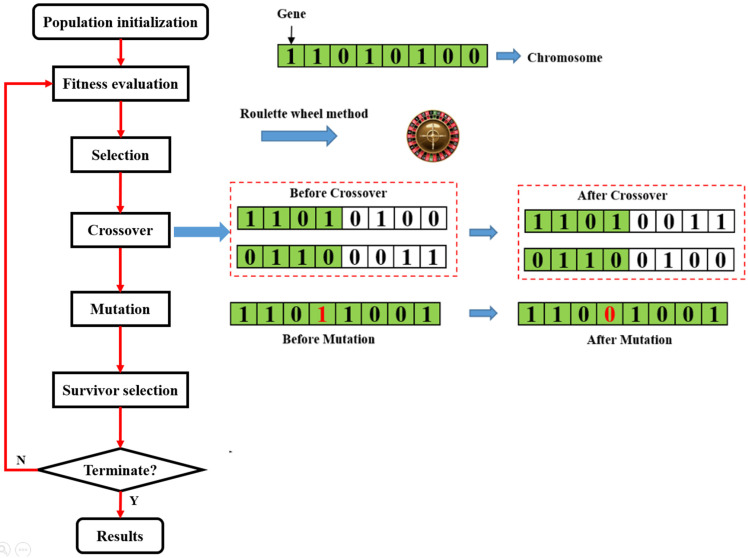
Flowchart of the genetic algorithm.

The development of the Genetic Algorithm (GA) was inspired by natural evolution and was widely used in engineering optimization due to its ability to solve optimization problems with unknown geometry of the searching space [90,91]. Each candidate is represented by a chromosome that can be altered or mutated. Generally, a chromosome is represented in a binary string of 0 s and 1 s. The length of a string is determined by the calculation scope and precision. In the process of evolution, a population of individuals are randomly generated. In each iteration, the objective function of the optimization problem to be solved is used to assess the fitness of each chromosome in the generation. The chromosomes with better objective values are randomly selected (usually by a roulette-wheel method) to yield offspring. The genes between two chromosomes are exchanged as per crossover schemes, including single-point crossover, uniform crossover, and multi-point crossover [92]. Gene mutation also occurs in this procedure, i.e., the binary code of a gene is changed from 0 to 1 or vice versa at a low probability. Finally, the new generation will replace part of the old population. The evolutionary process stops when either the maximum iteration number or a satisfactory fitness level is reached. A flowchart of GA is shown in Figure 9.

##### Shuffled Frog Leaping Algorithm

**Figure 10:**
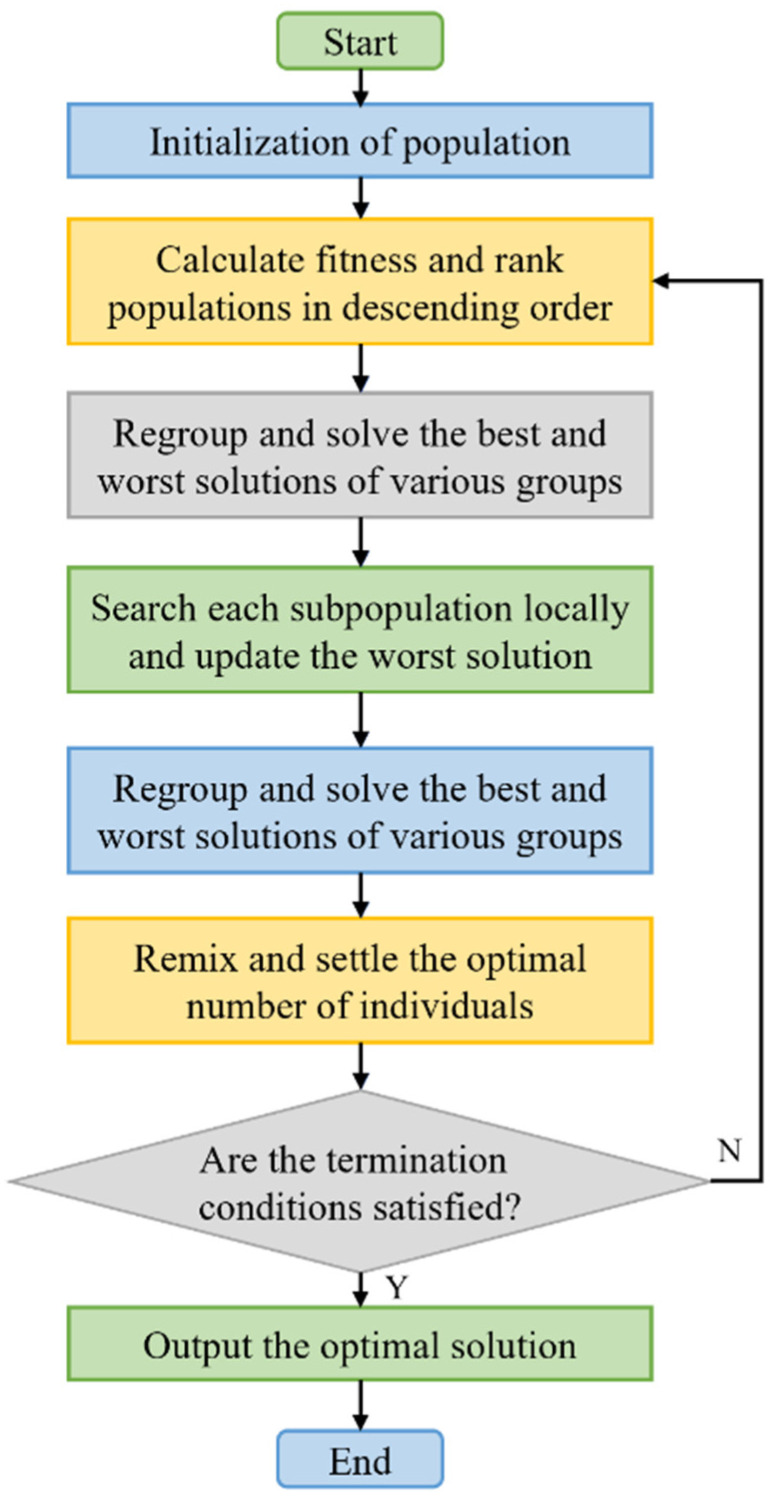
Flowchart of the shuffled frog leaping algorithm.

The shuffled frog leaping algorithm (SFLA) is a metaheuristic algorithm which achieves the goal of population optimization by simulating the process of frog foraging. In a limited space, frogs are distributed in different positions according to certain rules, which is called the initial position. Frogs independently search in groups to form different small groups, called subpopulations, and then they use their own personalized information to move in the direction of food in their respective areas to complete the position update. All the subpopulations that have completed the search are reorganized, and the frogs exchange information with each other, and then regroup. The above process is repeated until the frogs find the most appropriate food source. A flow chart of SFLA is shown in Figure 10.

##### Artificial Bee Colony Algorithm

The Artificial Bee Colony (ABC) algorithm is an optimization algorithm inspired by bee colony behavior; the optimization process of this algorithm is as follows.

**Figure 11:**
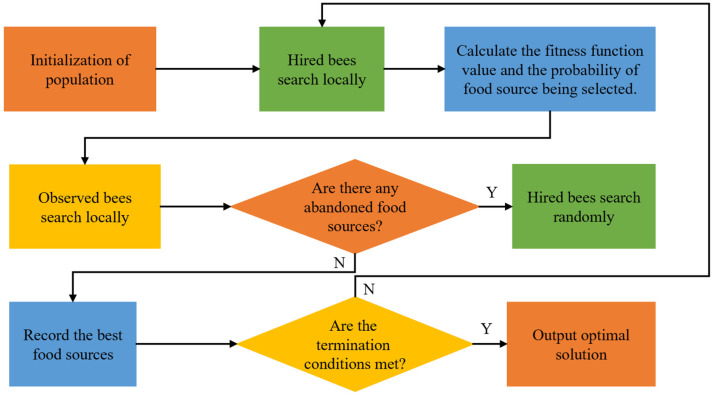
Flowchart of the Artificial Bee Colony algorithm.

(1) Initialize the population and generate N initial solutions of D dimensions randomly. X represents a bee population, and $X=(X_{1},X_{2},X_{3},\cdots X_{N})$, the resulting random feasible solution is defined as
  (28) $$X_{i}^{j}=X_{\min}^{j}+rand*(X_{\max}^{j}-X_{\min}^{j})$$
  where j represents a component of the dimension, calculates the fitness function values of each dimension, and defines the top half of bees with fitness function values as the initial hire bee population.
(2) Each hired bee will generate a new food source around the existing food source. The generation rules are defined as follows:
  (29) $$newX_{i}^{j}=X_{i}^{j}+\delta (X_{i}^{j}-X_{k}^{j})$$
  (30) $$P\left\{T_{s}(X_{i},newX_{i})=newX_{i}\right\}=\left\{ \begin{array}{l} 1\\ 0 \end{array}\right.$$
  where k, i, and δ are randomly generated numbers, k≠i and δ∈[−1,1]. After the new food source is generated, it is necessary to compare and evaluate the new food source and the old food source. If the new food source is better than the old food source, the old food source will be replaced with the new food source, otherwise the old food source will remain unchanged.
(3) The observed bees choose whether to follow the hired bees from the food information transmitted by them, so it is a probability problem. The definition of this probability is as follows:
  (31) $$p_{i}=\frac{f(x)}{\sum_{i=1}^{N}f(X_{i})}$$
  where $f(X_{i})$ represents the fitness function value of the i th food source, and it is defined as follows:
  (32) $$f(X_{i})=\left\{ \begin{array}{l} \frac{1}{1+f_{i}},f_{i}>0\\ 1+abs(f_{i}),f_{i}<0 \end{array}\right.$$
  where $f_{i}$ represents the objective function corresponding to the i th food source.
(4) If a food source is still not improved after iteration of the set number of cycles, the corresponding hired bee will become an observed bee, and a new food source will be generated to replace the abandoned food source:
  (33) $$X_{i}^{j}(n)=X_{\min}^{j}+rand(0,1)*(X_{{}_{\max}}^{j}-X_{\min}^{j})$$
  The generation of new food sources is conducive to ensuring the diversity of the population and improving the probability of finding the optimal solution. A flow chart of the ABC algorithm is shown in Figure 11.

#### 3.4.2. Single-Objective Optimization of Mixture Proportions of Cementitious Materials

Much research has been conducted into the single-objective mixture optimization of cementitious materials using ML and metaheuristic optimization algorithms. Usually, most of the research focuses on obtaining a mixture with the minimum cost at a specified UCS. Cheng et al. applied SVM and GA to optimize the mixture proportions of high-performance concrete [93]. This approach can minimize concrete cost at a given UCS. Other ML models and metaheuristic optimization algorithms are also employed to find minimum mixtures of different types of cementitious materials at specific cost. For example, Golafshani and Behnood employed biogeography-based programming to optimize the mixture proportions of silica-fume concrete [42]. They successfully found the minimum cost of a mixture with the UCS satisfying the requirement. Lee used ANN and a harmony search algorithm to optimize the cost and UCS of high-performance concrete [94]. Yeh achieved the lowest cost at a given UCS and slump of high-performance concrete using ANN and optimization technologies. Although three objectives (i.e., cost, UCS, and slump) are considered in their research, it is still single-objective mixture optimization. The Pareto front of the three objectives cannot be obtained by their method [95]. To solve multi-objective mixture optimization problems, the previous single-objective optimization method must be extended to MOO algorithms.

#### 3.4.3. Multi-Objective Optimization

##### Definition of the MOO Problem

We can use simple operators to find the global optimum of a single-objective optimization problem. However, for MOO problems, these operators are not applicable. Before solving an MOO problem, the following definitions should be given [96]:

**Definition** **1.** *Minimization problem: The minimization problem is defined as*

$$\min F\left(x\right)={\left[f_{1}\left(x\right),\;f_{2}\left(x\right),\ldots ,f_{k}\left(x\right)\right]}^{T}$$

(34) $$Subject\;to:\{\begin{array}{c} g_{j}\left(x\right)\geq 0,\;j=1,2,\ldots ,t\\ h_{j}\left(x\right)=0,\;j=1,2,\ldots ,m\\ l_{j}\leq x_{j}\leq \mu_{j},\;j=1,2,\ldots ,p\; \end{array}$$

*where F(x) is the objective function containing k objectives; t, m, and p are the numbers of inequality constraints, equality constraints, and variables, respectively; $g_{j}\left(x\right)$ and $h_{j}\left(x\right)$ are the j-th inequality and equality constraints, respectively; and [$l_{j},\mu_{j}$] denotes the boundaries of the j-th variable.*

**Definition** **2.** *Pareto dominance: If ∀i∈{1,2,…,D}: $u_{i}\leq v_{i}$, and ∃i∈{1,2,…,D}:$\;u_{i}<v_{i}$, then, vector u dominates vector v (u≺v), $u,v\in {\mathbb{R}}^{D}$.*

**Definition** **3.** Pareto set and Pareto front: For a given F(x), assume that $\Gamma \subset S_{x}$ is a vector set. If there is no x∈Γ that satisfies F(x) ≺ F(x^*^), then $x^{*}\in \Gamma$ is called a Pareto solution. The Pareto set is given by

(35) $$P_{\Gamma }=\{x^{*}\in \Gamma |\neg \exists x\in \Gamma :F\left(x\right)≺\;F\left(x*\right)\}$$

*where ¬∃ represents nonexistence. The Pareto front is defined as*

(36) $$PF=\{F\left(x\right)\in S_{y}|x\in P_{\Gamma }\}$$

**Definition** **4.** Pareto optimal set: For a given MOP F(x), $x^{*}\subset S_{x}$ is a Pareto optimal solution if there exists no feasible solution x satisfying F(x) ≺ F(x^*^). The Pareto optimal set ∧ is defined as

(37) $$\wedge =\{x^{*}\in S_{x}|\neg \exists x\in S_{x}:\;F\left(x\right)≺\;F\left(x^{*}\right)\}$$

**Definition** **5.** *Pareto optimal front: For a given F(x) and a Pareto optimal set* ∧, the Pareto optimal front is defined as

(38) $$PF^{*}=\{F\left(x\right)\in S_{y}|x\in \wedge \}$$

##### Construction of MOO Problems Using the Weighted Sum Method

To extend single-objective metaheuristic optimization algorithms into MOO algorithms, the Weighted Sum method combines multiple multi-objective functions into one objective and is widely used [87]:

(39) $$F\left(x\right)=\alpha_{1}f_{1}\left(x\right)+\alpha_{2}f_{2}\left(x\right)+\cdots +\alpha_{p}f_{p}\left(x\right)$$

where $\alpha_{i}$ is the weighting coefficient. 

The single objective problem using the Weighted Sum method is not strictly equivalent to the MOO problem due to the arbitrary selection of the weighting coefficients. In addition, the weighted sum function can be constructed in many ways (though the linear function is the mostly widely used). For instance the following quadratic form can be used:

(40) $$F\left(x\right)=\alpha_{1}f_{1}^{2}\left(x\right)+\alpha_{2}f_{2}^{2}\left(x\right)+\cdots +\alpha_{p}f_{p}^{2}\left(x\right)$$

### 3.5. Multi-Objective Optimization of Mixture Proportions of Cementitious Materials

As stated above, multiple competing objectives are involved in mixture optimization problems. MOO methods have attracted more and more interest recently. Several scholars have explored multi-objective metaheuristic algorithms combined with ML models for MOO of mixture proportions of cementitious materials. Baykasoğlu et al. proposed a MOO model for mixture optimization of high-strength concrete [97]. They considered three objectives including UCS, slump, and cost which were modelled using regression analysis, ANN, and Gene Expression programming. The Genetic Algorithm was used as the optimization algorithm to optimize mixture proportions. This paper introduced the concept of Pareto optimality in concrete mixture optimization. The authors treated the slump as constraints and hence this study is actually a bi-objective mixture optimization. Zhang et al. proposed a framework for multi-objective mixture optimization for cementitious materials [6]. They optimized mixture proportions of plastic concrete considering three objectives: UCS, cost, and slump using ML models and multi-objective PSO. They also compared different ML models in the prediction of different properties of cementitious materials and showed that BPNN is more accurate in continuous data (e.g., UCS), while RF performs better in more discrete data (e.g., slump). In addition, the decision-making method—the technique for order preference by similarity to an ideal solution (TOPSIS)—was first introduced to select final solutions in the Pareto front. It should be noted that more objectives (e.g., strength, workability, cost, environmental) should be considered in future work, though it is difficult to represent more than three objectives in the Pareto front.

## 4. Future Prospects

### 4.1. Considering More Properties of Components for Modelling Objectives

The objectives of mixture design are influenced by complex chemical and physical interactions between the components of cementitious materials. For example, the different properties of aggregates, such as size, shape, chemical composition, grading, absorptivity, and surface texture should be considered. The interfacial bonds between the mortar and aggregates are impacted by these properties, which, in turn, affect the strength of cementitious materials [98]. In addition, the objectives of concrete (e.g., mechanical, environmental, cost, workability) are also influenced by the properties of supplementary cementitious materials like silica fume, slag, and fly ash, such as pozzolanic reactivity, chemical variability, and fineness [99]. Furthermore, the additional processing steps and original industrial source significantly affect the mineral composition and fineness of the properties of these supplementary cementitious materials.

The generalization ability of the MOO models increases with increasing volumes of data. It is well known that the properties of field-placed concrete are highly variable due to the variability of the conditions of the jobsite. Hence, environmental conditions such as inclement weather, humidity, and temperature should be considered. The above-mentioned models only consider material composition for modelling objectives of cementitious materials in a laboratory. Such variabilities cannot be found in laboratory data and thus the current ML models should be extended by considering both the effects of materials and environments.

### 4.2. Developing New Metaheuristic Optimization Algorithms

A metaheuristic optimization algorithm is used to search for optimal mixture proportions based on the previously established ML model. Nonetheless, multi-objective mixture optimization problems are complex, and metaheuristic algorithms including the well-known PSO and GA may not achieve a good performance if they are trapped in local optima when most of the individuals in a population have similar structures [100]. For instance, the searching efficiency of GA will be eliminated if the population has converged, as new off spring cannot be produced by the crossover of almost identical chromosomes. It then only randomly and slowly searches for new domains because of the mutation process. 

For mixture optimization problems, a large amount of time is usually required because of the stochastic characteristics of the searching approaches of metaheuristic algorithms based on swarm intelligence [101]. Therefore, it is imperative to develop robust and efficient metaheuristic algorithms that can obtain optimal solutions under the conditions of limited financial, time, and material resources for mixture optimization. Recently, the beetle antennae search algorithm (BAS) was proposed based on individual intelligence [102]. This algorithm uses an individual (a beetle) rather than a swarm to search, and hence the calculation time is significantly reduced. Furthermore, this method is easy to implement with simple code and by using a specific step size strategy it is less likely to be trapped in local optima [103]. Inspired by the previously successful application of BAS to solve complicated engineering problems, this review proposes a multi-objective BAS (MOBAS) for solving multi-objective SFC mixture optimization problems.

#### 4.2.1. Basic Beetle Antennae Search Algorithm 

**Figure 12:**
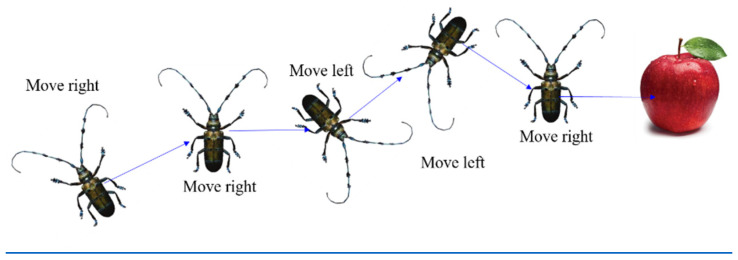
The foraging behavior of the beetle [104].

**Figure 13:**
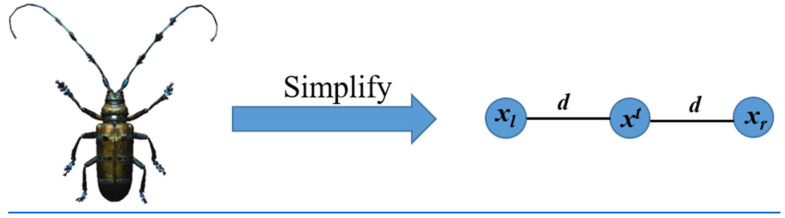
Simplified beetle model [104].

The Beetle Antennae Search (BAS) algorithm mimics the beetle’s foraging behavior [102]. The beetle searches for food using its two antennae. When the concentration of odor on the left-antennae is higher, the beetle moves to the left; otherwise it moves to the right, as shown in Figure 12. The beetle is simplified to develop the algorithm as shown in Figure 13. In this model, $x_{l}$ and $x_{r}$ represent a position on the left-antennae side and right-antennae side, respectively; $x^{i}$ denotes the position of the beetle at the ith time instant (*t* = 1, 2…); and *d* is the distance between the two antennae.

The beetle searches for food in a random direction and we define a random search vector as

(41) $$b=\frac{rand\;\left(k,1\right)}{\left\|rand\;\left(k,1\right)\right\|}$$

where *rand* is a random function and *k* denotes the dimension of the searching space. The position vector of the antennae top can then be written as

(42) $$x_{r}^{i}=x^{i}+d^{i}b$$

(43) $$x_{l}^{i}=x^{i}-d^{i}b$$

The position vector of the beetle can be formulated using the following iterative equation:

(44) $$x^{i}=x^{i-1}+\delta^{i}bsign\left(f\left(x_{r}^{i}\right)-f\left(x_{l}^{i}\right)\right)$$

where δ is the step size of the beetle. To avoid local optima, the following step size and antennae length updating strategy can be used:

(45) $$d^{i}=0.95^{i-1}+0.01$$

(46) $$\delta^{i}=\delta^{i-1}$$

**Figure 14:**
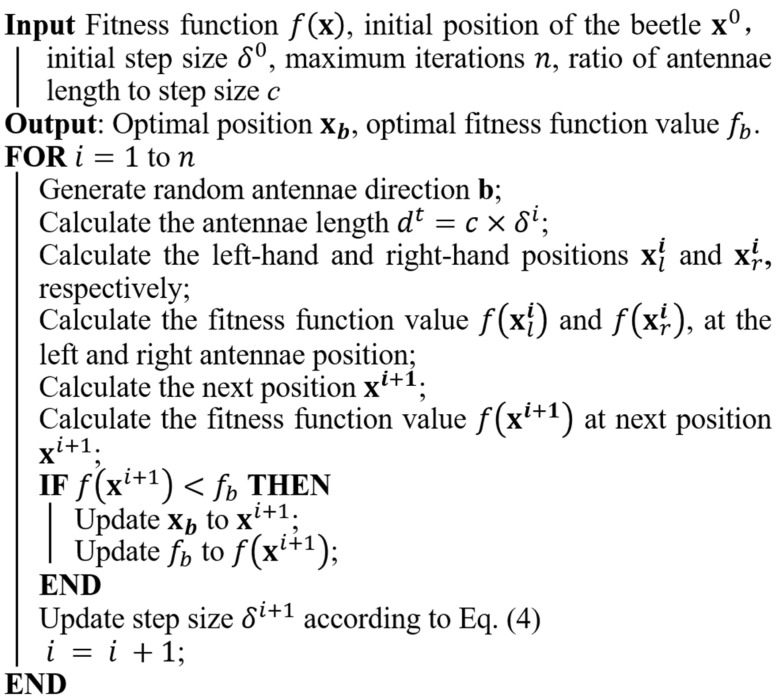
Pseudocode of BAS [105].

The pseudocode of BAS is shown in Figure 14.

#### 4.2.2. Improving Beetle Antennae Search using Levy Flight and Self-inertia Weight

To reduce the risk of trapping in local optima, the step size of the beetle can be adjusted by Levy flight and self-inertia weight. The improved BAS has been used in the prediction of concrete properties and the design of concrete mix in past studies [105,106,107,108,109,110]. It should be noted that for traditional BAS used in concrete design, the beetle step size is constant or decreases in each iteration. Adopting this step adjustment strategy can cause some problems. If the step size is too small, the BAS algorithm may converge slowly or fall into the local optimal state. However, if the given step size is very large, the global optimality may be skipped and the result may oscillate. Therefore, Levy flight and self-inertia weights should be used to adjust the step size of BAS.

(1) Levy flight

Many researchers have adopted Levy flight to improve the searching efficiency of swarm-based metaheuristic optimization algorithms [111,112,113]. When the beetle is trapped in a local minimum, the Levy flight will be triggered to enlarge the step size of the beetle:

(47) $$\delta^{\left(i\right)}=\alpha \left|Levy\right|⊗\delta^{\left(i-1\right)}$$

where α is a randomization parameter; α∈[0, 1]; ⊗ is entrywise multiplications; |Levy| represents a Levy distribution with an infinite variance and an infinite mean:

(48) $$Levy\sim u=t^{-\lambda },(1<\lambda \leq 3)$$

The Levy flight is triggered when the difference between the adjacent two fitness values ($f^{\left(i\right)}\;$ and $f^{\left(i-1\right)}$) is smaller than the difference between the historical worst and best fitness values ($f_{w}$ and $f_{b}$), which can be expressed as follows:

(49) $$\left|f^{\left(i\right)}-f^{\left(i-1\right)}\right|<\mu \left(f_{w}-f_{b}\right)$$

where μ is a coefficient.

(2) Self-adaptive inertia weight

The authors use a monotonically decreasing function to implement the self-adaptive inertia weight:

(50) $$\delta^{i+1}=\eta^{i}\times \delta^{i}$$

where $\delta^{i}$ is the step size at the current position and $\eta^{i}$ is the self-adaptive inertial weight which can be calculated as follows:

(51) $$\eta^{i}=\left(1-\alpha \right)0.95+\alpha \frac{f_{w}^{i}-f^{i}}{f_{w}^{i}-f_{b}^{i}}$$

where $f^{i}$ is the fitness function at the current position; $f_{w}^{i}$ and $f_{b}^{i}$ represent the worst and the best fitness values, respectively; α is a hyper-parameter to trade off between the two items, where the first item ((1−α)0.95) indicates the inertial weight and the second item ($\alpha \frac{f_{w}^{i}\;-\;f^{i}}{f_{w}^{i}\;-\;f_{b}^{i}}$) represents the self-adaptive property. Algorithm 1 shows the improved BAS algorithm by using Levy flight and self-adaptive inertia weight. 

**Algorithm 1**: Modified BAS**Input**: Fitness function $f(x^{t})$, initial position of the beetle $X^{0}$, initial step size $\delta^{0},$ max imum iteration number n, ratio of antennae length to step size c, attenuation coefficient of step size η.**Output**: Optimal positions $X_{b}$, optimal fitness function value $f_{b}$.**FOR** i=1 to n     Generate random antennae direction b;     Calculate the antennae length $d^{i}=c\times \delta^{i}$;     Calculate the left-hand and right-hand positions $x_{l}$ and $x_{r}$;     Calculate the fitness function value $f(x_{l})$ and $f(x_{r})$ at the left and right antennae position;      Calculate the next position $x^{i}$;     Calculate the fitness function value $f(x^{i+1})$ at next position $x^{i+1}$;     **IF** $f(x^{i+1})<f_{b}$     **THEN** Update $x_{b}$ to $x^{i+1}$; Update f(b) to $f(x^{i+1})$;     **END**     Update step size $\delta^{i+1}$ using Equation (11);     **IF** $\left|f(x^{i+1})-f(x^{i})\right|<\mu (f_{w}-f(b))$     **THEN** Update the size $\delta^{i+1}$ using Levy flight according to Equation (9);
     **ELSE** Update the size $\delta^{i+1}$ according to Equation (8)     **END**      i=i+1
**END**


#### 4.2.3. Multi-Objective Beetle Antennae Search Algorithm 

The previously mentioned Weighted Sum method can be used to extend the basic BAS to MOBAS. The pseudocode for MOBAS is shown below as Algorithm 2.

**Algorithm 2:** MOBAS**Input**: Fitness function $F={\left[f_{1}\left(x_{j}^{i}\right),\ldots ,f_{k}\left(x_{j}^{i}\right),\ldots ,f_{K}\left(x_{j}^{i}\right)\right]}^{T}$, initial position of the beetle $X^{0}=\left\{x_{1}^{0},\ldots ,x_{j}^{0},\ldots \right\}$, initial step size $\delta^{0}=\left\{\delta_{1}^{0},\ldots ,\delta_{j}^{0},\ldots \right\},$ maximum iteration number N, ratio of antennae length to step size c and step size attenuation coefficient α.**Output**: M optimal Pareto positions (non-dominated solutions) $X_{PF}=\left\{x_{PF,1},\ldots ,x_{PF,m},\ldots ,x_{PF,M}\right\}$m = 1**;****WHILE** (m≤M)     Calculate the random weight of each objective $\Omega =\left[\varpi_{1},\ldots ,\varpi_{k},\ldots ,\varpi_{K}\right]$, and normalized with $\varpi_{k}=\sum_{k=1}^{K}\varpi_{k}$;     **FOR** i=1 to N
     Generate random antennae direction $b^{i}$;     Calculate the antennae length $d^{i}=c\times \delta_{m}^{i}$;
     Calculate the left-hand and right-hand positions $x_{l}^{i}$ and $x_{r}^{i}$;     Calculate the weighted sum function value $\Phi \left(x_{l}^{i}\right)$ and $\Phi \left(x_{r}^{i}\right)$ at the left and right antennae position with Φ(x)=Ω·F;      Calculate the next position $x^{i+1}=x^{i}+\delta^{i}$;     Calculate the weighted sum function value $\Phi \left(x^{i+1}\right)$ at next position $x^{i+1}$;     **IF** $\Phi \left(x^{i+1}\right)<\Phi_{b}$ **THEN**     Update $x_{b}$ to $x^{i+1}$;     Update $\Phi_{b}$ to $\Phi \left(x^{i+1}\right)$;     **END IF**     Update step size $\delta^{i+1}=\alpha \delta^{i}$;     **END FOR**     **IF**
$x_{b}$ satisfy all the constraints     **IF**
$x_{b}$ is not dominated by $X_{PF}$**, THEN**     Update $X_{PF}=X_{PF}\cap x_{b}$;     Update m=m+1;     **END IF**     **FOR**
$X_{PF,t}$
**IN**
$X_{PF}$     **IF**
$x_{b}$ dominates $x_{PF,t}$, **THEN**     Update $X_{PF}=X_{PF}-x_{PF,t}$;     Update m=m−1                 **END IF**              **END FOR**     **END IF**
**END WHILE**


### 4.3. Incorporating Many Objectives

Currently, only one or two objectives (usually UCS and cost) are considered in the optimization studies. However, in real applications, many objectives are important. Thus, in future work, it is necessary to develop many-objective optimization models that are able to consider the trade-offs between a large number of competing objectives. For example, fresh properties of cementitious materials (e.g., slump and set time) should be incorporated. As an indicator of workability, slump is mostly influenced by water-to-cement ratio, gradation and shape of aggregates, superplasticizer, and air entraining agents [114], while set time is significantly affected by water-to-cement ratio, cement fineness, cement type, types and amount of supplementary cementitious materials, and superplasticizers. Therefore, input variables should be carefully selected for different objectives. In addition, when optimizing the mixture proportions of functional cementitious materials, specific objectives need to be taken into consideration, such as the permeability of pervious concrete, the density of lightweight concrete and the flexural strength of fiber reinforced concrete.

In addition, it is known that in the cement production process, approximately 0.9 ton of CO_2_ is emitted for each ton of cement [115]. The cement industry contributes approximately 5–7% of global CO_2_ [116]. Therefore, the greenhouse gas emissions are particularly high for concrete production due to the extensive use of cement. It is natural for engineers and researchers to consider environmental objectives for mixture optimization of cementitious materials. Changing the decision variables is an effective way to reduce the impact of cementitious materials on the environment. Some researchers have used supplementary cementitious materials (e.g., silica fume, blast furnace slag, and fly ash) to replace part of the cement in order to reduce CO_2_ emission [117,118,119]. Other researchers have applied recycled aggregate to replace partial or whole natural sand, or natural coarse aggregates in concrete to reduce the depletion of non-renewable energy resources [120,121]. Therefore, it would be very useful to develop many-objective mixture optimization models that can trade off between many objectives (e.g., mechanical properties, cost, environmental impact, and durability) to optimize mixture proportions containing various decision variables.

Other challenges that exist in many-objective mixture optimization are how to visualize the data with many objectives and how to select a final solution on the resulting Pareto front. To visualize the data, data visualization techniques, such as parallel coordinates, can be used to visualize and understand the solutions on the Pareto front [122]. For the selection of non-dominated solutions on the Pareto front, it is common to select the final mixture based on engineering requirements; however, multi-criteria decision selection methods such as TOPSIS (Technique of Order Preference Similarity to the Ideal Solution) can be applied [123]. 

## 5. Conclusions

Currently, much of the research literature focuses on single-objective mix optimization, such as finding the minimum cost of a mix for a particular UCS. However, the hybrid optimization of modern cementitious materials needs to meet multiple objectives simultaneously. These objectives are usually competing with each other, so the ML-based MOO model and swarm intelligence-based multi-objective optimization algorithm is applied to find the Pareto front of the multi-objective hybrid optimization problem.

In this paper, the common methods of mixture optimization (experimental method, Taguchi method, and response surface method) are reviewed. Then, the formulation of the cementitious material mixture optimization problem is discussed, including the determination of decision variables, application constraints, modeling objectives, and the development of metaheuristic optimization algorithms. The single-objective hybrid optimization problem is realized by the single-objective metaheuristic optimization algorithm, while for the multi-objective hybrid optimization problem, it is necessary to extend the single-objective metaheuristic optimization algorithm to the multi-objective metaheuristic optimization algorithm.

The future of cementitious material mixing optimization has also been prospected. By increasing the amount of data and considering more influencing variables, such as environmental factors and components, the generality of the existing MOO model is improved. Additional objectives should be considered when producing cementitious materials with improved working performance, strength, durability, reduced costs, and minimal environmental impact. Therefore, the development of computationally efficient robust MOO models will continue to make progress in the hybrid optimization field. 

This study sums up the mixture optimization of cementitious materials using machine learning and metaheuristic algorithms and it can be employed by engineers and researchers who want to optimize the mixture proportions of cementitious materials. However, it should be noted that more possible multi-objective optimization algorithms may be used for cement-based material design in the future, so more studies based on different algorithms will be carried out, and a more extensive literature review study should be carried out at that time. In addition, the reliability comparison between different algorithms should be paid more attention to in future research. Other challenges will also need to be addressed in the future, such as how to visualize data with multiple objectives and how to choose the final solution on the acquired Pareto front.

## Figures and Tables

**Table 1:** Examples of decision variables for mixture optimization of cementitious materials.

Decision Variable	Type
Cement content	Continuous
Water content	Continuous
SCMs content	Continuous
Aggregate content	Continuous
Superplasticizer content	Continuous
Curing age/temperature	Continuous
Cement type	Discrete
SCMs type	Discrete
Aggregate size	Discrete

## Data Availability

The data presented in this study are available on request from the corresponding author.
